# The relationship between Hikikomori risk factors and social withdrawal tendencies among emerging adults—An exploratory study of Hikikomori in Singapore

**DOI:** 10.3389/fpsyt.2022.1065304

**Published:** 2022-12-20

**Authors:** Patrick K. F. Lin, Andrew, Alethea H. Q. Koh, Kongmeng Liew

**Affiliations:** ^1^School of Social and Health Sciences, James Cook University, Singapore, Singapore; ^2^Institute for the Future of Human Society, Kyoto University, Kyoto, Japan; ^3^Graduate School of Science and Technology, Nara Institute of Science and Technology, Ikoma, Japan

**Keywords:** Hikikomori, social withdrawal tendencies, support from friends, DASS-21, emerging adults

## Abstract

**Introduction:**

Once a localized Japanese phenomenon, Hikikomori-type social withdrawal has since been observed globally in increasing numbers. However, there is a lack of research about Hikikomori in Singapore. Consequently, local variations of Hikikomori may differ from past research in Japan. Drawing on associations found in international and Japanese Hikikomori research, we explored some variables relevant and generalizable to the Singaporean context. Specifically, we examined the relationships between (1) Hikikomori risk factors, (2) social withdrawal tendencies, (3) depression and anxiety, (4) connections with family and friends, and (5) employment status.

**Methods:**

In a cross-sectional survey study (*N* = 416; *M*_*age*_ = 24.90, *SD*_*age*_ = 4.79; females = 236, males = 177, undisclosed = 3), participants were provided a Qualtrics link and asked to complete a questionnaire comprising the NHR scale, LSNS-6, DASS-21, ERQ, and HQ-25.

**Results:**

We found that (a) Hikikomori risk factors positively correlated with social withdrawal tendencies and depression and anxiety but negatively correlated with support from family and friends, (b) high Hikikomori risk factors predicted high social withdrawal tendencies, (c) support from friends (one of the psychosocial factors) predicted social withdrawal tendencies together with the Hikikomori risk factors, and (d) social withdrawal tendencies moderated the relationship between Hikikomori risk factors and depression among the emerging adults in Singapore.

**Conclusion:**

The current research findings serve as a basis for future Hikikomori research in Singapore.

## 1 Introduction

Hikikomori is a sociocultural mental health phenomenon where individuals experience severe forms of social withdrawal, which causes distress to themselves and others who care for them (1). Several countries, particularly in East Asia, have observed the growing prevalence of Hikikomoris in their populations. Yet, there are slight differences in terms used to describe this phenomenon. For example, in China, Hong Kong, and Singapore, Hikikomoris are called “hidden youth,” and in South Korea, “socially withdrawn youth” (2).

In the Hikikomori Round Table and Regional Symposium (HRTRS), clinicians from Japan, Hong Kong, South Korea, China, and Singapore, reached a consensus on the definition of Hikikomori, which is conceptualized with these core features: (1) Hikikomori refers to emerging adults who are physically isolated at home for 3–6 months or more, (2) they showcase psychological detachment from the world by being socially isolated, which could cause psychiatric comorbidities such as depression and anxiety, and (3) they might have experienced distress as their daily functioning are impaired (for example, daily tasks, such as buying food, are avoided to avoid interactions with others; (2). While more research would be needed to examine the degree of manifestation of each feature in different cultures, these features seem to suggest that Hikikomori-linked behavior may not be readily observable. Accordingly, diagnoses of Hikikomori may not be limited to the individual’s detachment from others—in some cases, Hikikomori individuals could still function according to social expectations, but isolate themselves psychologically and emotionally.

The need to conduct focal research on Hikikomori is further exacerbated by studies considering individuals displaying Hikikomori-linked features as having avoidant personality disorder, social anxiety disorder, agoraphobia, or depression (3, 4). While it is important not to blur these concepts together as their outcomes and associated coping mechanisms would be different, it remains unclear where the line is between such disorders and Hikikomori-linked features. Considering these circumstances, we conceptually separate Hikikomori into two distinct aspects: (1) *Hikikomori risk factors* and (2) *social withdrawal tendences*. While Hikikomori risk factors might reflect economic factors and societal values (etc., beliefs or views about the self and society, access to social support) in creating a perception of marginalization and hence withdrawal risk, social withdrawal tendencies relates more to the individual’s downstream situation, and actual withdrawal-linked tendencies and behavior. In other words, risk factors relate to the context and affordances of the situation that makes social withdrawal seem favorable to the individual, who might then start to display social withdrawal tendencies. For example, an individual with strong Hikikomori risk factors may show up for work regularly, but feel like they do not fit in with society and still desire to withdraw despite not being able to (5). On the surface, the individual might not show high social withdrawal tendencies, at least not enough to be diagnosable as Hikikomori, but conceptually, such Hikikomori risk factors nevertheless shares several common underlying psychological mechanisms with social withdrawal tendencies. We employ this distinction particularly in the examination of the Hikikomori phenomenon in Singapore, given that Singaporean culture, and social structures (etc., small land area and the difficulty of moving out) may have unique affordances for the manifestation of Hikikomori-linked behavior (such as high Hikikomori risk factors without social withdrawal tendencies).

Hence, an examination of Hikikomori risk factors as separate from social withdrawal tendencies is especially important for the Singapore context; if the unique societal structure does not allow for the manifestation of social withdrawal tendencies, Hikikomori risk factors may be a better informant of the actual Hikikomori-related prevalence in Singapore. In other words, how much do individuals want to or are at risk of social withdrawal regardless of whether they could. However, current measurements on Hikikomori risk factors may be more a reflection of cultural marginalization as an antecedent of withdrawal [see (6)] developed in a Japanese context. Hence, the measure itself may be culture specific, predicting Hikikomori behavior only in certain (Japanese) contexts [see (7)] and unknown in other contexts such as in Singapore. As such, given that Hikikomori is still under-researched in Singapore, we first aim to establish a deeper understanding of Hikikomori in Singapore through relating Hikikomori risk factors to social withdrawal tendencies in the current paper, and subsequently explore related constructs like employment status, family/friend connections, depression, and anxiety.

### 1.1 The origins of Hikikomori

The term “*Hikikomori”* was derived from two Japanese verbs: “*hiki”* means “to withdraw” and “*komori”* means “to be inside” (1, 8). It was coined by Japanese psychiatrist Tamaki Saito (1), who witnessed multiple youths in Japan in the 1990s exhibiting extreme social withdrawal behaviors.

During the 1990s, Japan underwent an economic “ice age” (9). Traditionally, youths were expected to excel academically to be recruited into major industries amidst stiff competition from peers (9). By being employed, youths, and their families believed that pride would be brought to their family’s name, and youths would be deemed successful in society’s eyes (9, 10). However, these youths were unprepared for the recession and globalization in the 1990s (11).

Recession and globalization in the 1990s led to change in the societal structure in Japan, where companies were focused on reducing labor costs and stopped hiring (10). Further, Japan’s seniority-based system ensured that senior employees’ jobs were preferentially protected (12), increasing competition among youths for a dwindling pool of secure jobs with average pay and causing distress, particularly among those who failed to secure a job (9, 10).

In addition, due to the interdependent cultural norms in Japan, youths are generally expected to respect and honor their seniors (e.g., parents, teachers, superiors), and uphold relational harmony (13). These youths were expected to subserviently accept the situation in the 1990s, while continuing to look for jobs to showcase their tenacity. For some who were unable to find jobs, this form of cultural marginalization caused some youth to deviate from their interdependent orientation, and withdraw from society (6, 10). By being reclusive, they could avoid further shame and disappointment from those around them (1, 8).

Since the 1990s, many have become accustomed to being reclusive. They found it daunting to look for jobs again, compete with their seniors and peers, and be placed into the “rat-race” of life (8). This “Hikikomori phenomenon” started to grow from 2000s (14, 15), as an avoidant reaction to highly pressurizing Japanese society, prompting many to play truancy and absenteeism. Young adults who have been working and felt tired of stiff competition for promotion decided to quit their jobs, stay at home and depend on their parents (9).

The Hikikomori phenomenon is still relevant and growing in Japanese society today. In a study from 2002 to 2006, Koyama et al. (14) surveyed 4,134 individuals in Japan. They reported a prevalence estimate that 1.2% of the general population in Japan would have experienced Hikikomori in their lifetime. They also reported that individuals with Hikikomori are 6.1 times higher risk of mood disorders (major depression, dysthymic depression, manic, and hypomanic episodes). Additionally, males are three times more susceptible to experiencing Hikikomori than females, and those aged 20–29 were the most common to experience Hikikomori (14). Research conducted by Japan’s Cabinet Office found that from 2000s to 2020s, more than 1 million individuals (age 15–64) with Hikikomori in Japan visited public health centers (16). Specifically, out of these 1 million individuals, 613,000 individuals between the ages of 40–64 have also met the criteria of Hikikomori. This suggested that Hikikomori might not be a condition that was only associated with youth (17). Furthermore, researchers found it was extremely difficult to diagnose Hikikomori individuals due to their social reclusion (14), and many researchers attempted to understand the prevalence of Hikikomori in Japan by administering clinical interviews to their families (14).

### 1.2 Quantifying Hikikomori risk factors and social withdrawal tendencies

Where many past attempts at quantifying Hikikomori adopted a fixed time-period criteria (e.g., 3–6 months), this one-size-fits-all approach may miss the nuances within Hikikomori populations. Accordingly, in the past decade, several scales have been developed to measure Hikikomori risk factors and social withdrawal tendencies in individuals. Of which, we focus on the NEET/Hikikomori risk (NHR) scale (6) as a measurement of Hikikomori risk factors and the 25-item Hikikomori Questionnaire [HQ-25; (18)] to measure social withdrawal tendencies. Uchida and Norasakkunkit (6) proposed that Hikikomoris should be viewed as a spectrum: Hikikomoris may cope with societal pressures by not “chasing” after typical social narratives (e.g., full-time employment or studies in a good school), but may choose to live an alternate lifestyle, for instance, only take on part-time working or be an independent artist. They focus on living differently from the social norm and fulfilling the idea of living in an alternative scene (e.g., underground and indie). Similarly, they may actively adopt a NEET (Not in Employment, Education, or Training) lifestyle (1). These are individuals who have actively chosen not to be employed, nor to be in a school system or get some training for a prolonged period (1). On a spectrum, some NEETs individuals may not be Hikikomori as they could be pursuing a socially active lifestyle. Although not working, studying, or in training, they are capable of having close relationships with their immediate families, relatives, and friends (1, 10). Other more severe cases may be completely isolated from family and friends, and live their life completely online. As such, the NHR scale allows researchers to identify individuals with Hikikomori risk factors through assessing the extent to which they perceive themselves as being marginalized by culture (6), by measuring their preference for the NEET lifestyle choice (e.g., “*I cannot find meaning in work*”), their internalization of failures to meet societal expectations (e.g., “*There are times when I think that I am not needed by society*”), and their apathy toward future prospects (e.g., “*I don’t quite know what I want to do in the future*”). Individuals high on NHR are more inclined toward social withdrawal, and past studies in Japan have shown that severe Hikikomori cases are also associated with high NHR scores (6). While the HQ-25 scale has some behaviorally oriented measurements (e.g., *“stay away from other people”*), it also assesses individual’s cognitions and emotions toward socialization (e.g., “*It is hard for me to join in on groups”*), isolation (e.g., “*I do not live by society’s rules and values”*), and amount of emotion support (e.g., “*There really is not anyone with whom I can discuss matters of importance*”). The HQ-25 allows researchers to accurately identify individuals with Hikikomori by assessing their actualized social withdrawal tendencies with the HQ-25’s sub-factors (i.e., socialization, isolation, emotional support). The outcomes will be able to help clinicians to provide interventions (18).

Varnum and Kwon (7) argue that the NHR scale measures may only measure Hikikomori risk factors in higher-income countries where residents have the financial affordance to withdraw, whereas in lower-resource societies, NHR may be a reflection of externalizing tendencies (e.g., youth delinquency). Uchida and Norasakkunkit (6) acknowledge this possibility, suggesting that NHR may only reflect Hikikomori arising from deviation from collectivistic cultural norms, as collectivistic societies may have stronger pressure on youths to fit predetermined cultural narratives. Accordingly, we needed to examine if NHR was also related to Hikikomori risk factors in Singapore, as a relatively higher-income and collectivistic society.

### 1.3 Hikikomori in Singapore

Multiple studies have shown that extreme social withdrawal is evident worldwide (19–26). However, it is not easy to ascertain its prevalence in most countries, as Hikikomori individuals would not seek psychological help due to their reclusiveness or they do not perceive themselves as dysfunctional (2).

In Singapore, due to the reclusive nature of Hikikomori, to our knowledge there have been no published studies on Hikikomori prevalence. We believe that similar to Hikikomoris elsewhere, Singaporean Hikikomoris are unlikely to interact with researchers and would not seek treatment (27). Even if they were to participate in studies, their responses might be invalidated or recorded as another disorder, due to the high levels of distress they may have experienced before being open to seek treatment (28).

However, there has been a growing interest in social withdrawal in local news media outlets (29). Most studies concerning Hikikomori in Singapore are conducted *via* retrospective information about Hikikomori experiences and assessing their risk tendencies (2, 5, 27). For instance, Bowker et al. (27) were interested in retrospective Hikikomori experiences of university students from Nigeria, Singapore, and United States. Singaporean students reported fewer Hikikomori experiences than Nigerian students but more Hikikomori experiences than American students (27).

Furthermore, male Singaporean students with past Hikikomori experiences reported higher levels of social anhedonia (disinterest and low drives for social interaction) than female Singaporean students with Hikikomori experiences and male Singaporean students without Hikikomori experiences (27). Moreover, Singaporean students displayed higher levels of depressive symptoms and anxiety than Nigerian and American students. This study suggests that Singaporean students do experience severe social withdrawal, which has corresponding effects on depressive and anxiety symptoms.

Another study with Singapore university students showed that Hikikomori risk factors were negatively correlated with self-esteem and wellbeing, and positively correlated with poor perceived social relationships and depression tendencies (5). While these findings showed consistencies with previous Japanese research, they could not conclude if Hikikomori risk factors would lead to withdrawal tendencies, which was a question we sought to address with the current paper.

### 1.4 Psychosocial and psychopathological issues associated with Hikikomori

#### 1.4.1 Psychosocial

Psychosocial factors are among the most studied contributing primary issue to the onset of Hikikomori (30), such as family and friends’ lack of social support (3). Research indicated that a weak relationship with family and friends would lead to social reclusion, which could be due to poor social skills (3) or fear of social judgment (12). At the family level, mid to high socioeconomic status, high academic expectations from families, over-protective parents that lead to co-dependent behaviors, and attachment issues (e.g., lack of physical or psychological care from their family members) would influence them to be reclusive (2) through creating strong social pressures. Within the school context, the risk factors consist of peer bullying, academic difficulties, and harsh discipline by teachers (2). These are observed more frequently in profiles of primary Hikikomori [who have no known co-morbidities; (30)]. In particular, Singapore has a relatively interdependent culture with a national focus on promoting community and familial ties, within a relative densely populated space. Consequently, individuals’ engagement to family and friends may be an especially important psychosocial risk factor in the Singaporean context through the affordances for Hikikomori tendencies from the support and expectations of others.

#### 1.4.2 Psychopathological

Hikikomoris with co-morbid psychopathologies are termed “secondary Hikikomoris” (30). Research has shown that individuals (and their family members) with depression, anxiety, poor stress management, poor emotional regulation, schizophrenia, obsessive-compulsive disorder, avoidant personality disorder, and autism spectrum disorder tend to be reclusive to feel safe from being judged by others (2, 14, 31). Furthermore, research has shown that youths with Hikikomori tend to develop gaming or internet addiction as it becomes a coping mechanism (2). 54.5% of the Japanese Hikikomori population are comorbid with psychological disorders, such as depression, which results in reclusiveness (14). Similarly, over 80% of Spain’s Hikikomori population had psychosis and anxiety as their common comorbid disorders (32). Some studies in Japan also showed that older Hikikomori individuals had anger and depression issues (33).

### 1.5 Aim of current study

As Liew et al. (5) established that NHR in Singapore seemed to somewhat mirror the Japanese context, we hypothesized that NHR (i.e., Hikikomori risk factors) would also be associated with social withdrawal tendencies in Singapore. As typical mean ages of Hikikomori are found within their 20s and are usually seen in their youth (14), we aimed to recruit participants within the age range of 18–35, following the age range defined for “emerging adults” by the National Youth Council of Singapore (34).

We then explored issues associated with Hikikomori risk factors and social withdrawal tendencies in the local Singaporean context. Based on past research, it would be rational to examine (a) connections from family and friends (psychosocial issues), and (b) depression, anxiety, and stress (psychopathological issues). With these variables, we aim to explore and build possible models to gain a preliminary understanding of how Hikikomori risk factors may lead to social withdrawal tendencies in Singapore. Furthermore, researchers have suggested that variables such as employment status, gender, age, and emotional regulation strategies could also contribute to the Hikikomori phenomenon, therefore, we also included them in the current research.

## 2 Materials and methods

### 2.1 Participants and design

The current study employed a cross-sectional survey approach. Based on the power calculation (with power of 0.90, effect size of small to medium, *f*^2^ = 0.05–0.10, and alpha of 0.05) with 6 predictors (i.e., social withdrawal tendency, Hikikomori risk factors) in a regression approach, it was recommended to obtain sample size between 181 and 322 for sufficient power. In the current study, we recruited 416 participants (*M*_*age*_ = 24.90, *SD* = 4.79). Within the 416 participants, 177 (42.5%) identified as males, 236 (56.7%) identified as females, and 3 (7%) identified as others. Participants were recruited from five local universities in Singapore and social media platforms (e.g., Facebook) through snowball sampling. There were 211 university students (50.7%) in the current study.

### 2.2 Materials

#### 2.2.1 NHR scale

The NHR scale is used to measure Hikikomori risk factors. It indicates good validity and reliability [Cronbach’s alpha is 0.88; (6)]. Participants completed a 27-item questionnaire, which measures three factors: Freeter (NEET) lifestyle preference, lack of self-competence, and unclear ambition for the future. Participants responded to each question on a 7-point Likert scale (*Completely Disagree* = 1 to *Completely Agree* = 7). Some questions such as “*I think that a person who does not work will become lazy*” were reverse scored. The sum score was used for analysis, where higher scores indicate higher levels of social withdrawal tendencies.

#### 2.2.2 HQ-25

The HQ-25, a 25-item questionnaire, is used to measure social withdrawal tendencies over the past 6 months. It indicates excellent validity and reliability [Cronbach’s alpha is 0.96; (18)]. Participants responded to each question on a 5-point Likert scale (*Strongly Disagree* = 0 to *Strongly Agree* = 4). Some questions such as “*I love meeting new people*” were reverse scored. The sum score was used for analysis, where higher scores indicate higher levels of social withdrawal tendencies.

#### 2.2.3 LSNS-6

Participants completed the LSNS-6, a 6-item questionnaire subset of the 12-item Lubben Social Network Scale (35). The LSNS-6 measures an individual’s social engagement with family and friends with a Cronbach’s alpha of 0.83 (35). Participants responded to each question (e.g., *How many relatives do you see or hear from at least once a month*) on a 6-point Likert scale (*none* = 0 to *nine or more* = 5). The sum score was obtained for analysis, where higher scores indicate higher levels of social engagement of family and friends.

#### 2.2.4 Depression, anxiety, stress scale—21 items (DASS-21)

The DASS-21 is a 21-item questionnaire, a subset of the original 42-item questionnaire, was used to measure the depression, anxiety, and stress levels of an individual for the past 1 week (36). Participants were asked to answer the questions that consist of the Depression scale (e.g., *I couldn’t seem to experience any positive feeling at all*), the Anxiety scale (e.g., *I was aware of dryness of my mouth*), and the Stress scale (e.g., *I find it hard to wind down*). All items were based on a 4-point Likert scale (*did not apply to me at all* = *0 to applied to me very much or most of the time* = *3*). The DASS-21 has been demonstrating excellent internal consistency with Cronbach’s alpha ranging from 0.87 to 0.94 (37). The scores for depression, anxiety, and stress were obtained by tallying the scores of each category and multiplying by 2, where higher scores indicate either higher levels of depression, anxiety, or stress.

#### 2.2.5 ERQ

The ERQ is used to measure how individuals would regulate their emotions: by changing the way they think about their emotions (reappraisal) or suppressing their emotions [suppression; (38)]. Participants completed a 10-item questionnaire (e.g., *I control my emotions by changing the way I think about the situation I’m in* for reappraisal; *I control my emotions by not expressing them* for suppression) with a 7-point Likert scale (*Strongly Disagree* = 1 to *Strongly Agree* = 7). The EQR has good internal consistency with a Cronbach’s alpha is 0.79 (38). The sum scores of reappraisal and suppression were obtained for the analysis, with higher scores indicate either higher levels of reappraisal or suppression.

### 2.3 Procedures

Participants were recruited *via* local private universities in Singapore and social media platforms (e.g., Facebook). Upon signing up, participants would be provided a Qualtrics link and directed to the information and consent page. Next, they would be asked some basic demographic information (e.g., gender, age, employment status etc.) before being tasked to complete a questionnaire comprising the NHR scale, LSNS-6, DASS-21, ERQ, and HQ-25. The survey took approximately 30 min and participants were then thanked for their participation once they completed the questionnaire. The study was approved by James Cook University Human Ethics Committees (H8001).

## 3 Results

### 3.1 Descriptive analysis

Table 1 provides an overview of the descriptive statistics for our sample.

**TABLE 1:** Demographic characteristics of the study sample (*n* = 416).

Demographic characteristics	*n* (%)
**Ethnicity**	
Chinese	327 (78.6)
Malay	34 (8.2)
Tamil	33 (7.9)
Eurasians	4 (1)
Others^a^	18 (4.3)
**Highest level of education achieved**	
Secondary school certification (N/O levels)	21 (5)
Institute of technical education (ITE) certification	6 (1.4)
Polytechnic diploma or a junior college “A” level certification	185 (44.5)
Undergraduate degree	163 (39.2)
Postgraduate diploma	24 (5.8)
Postgraduate degree (Masters or PhD)	17 (4.1)
**Disabilities**	
Yes	14 (3.4)
No	402 (96.6)
**Marital status**	
Married	47 (11.3)
Divorced	3 (0.7)
Separated	1 (0.2)
In a romantic relationship	131 (31.5)
Single	234 (56.3)
**Employment status**	
Full-time	149 (35.8)
Part-time	24 (5.8)
Contract/Temporary	14 (3.4)
Student	211 (50.7)
Unemployed	7 (1.7)
Others^b^	11 (2.6)
**Annual income**	
Less than S$25,000	245 (58.9)
S$25,000–S$49,999	98 (23.6)
S$50,000–S$99,999	56 (13.5)
S$100,000–S$199,999	12 (2.9)
S$200,000 and above	5 (1.1)
**Number of family members who stays in the same** **household as you (via birth/marriage)**	
0	11 (2.6)
1	43 (10.3)
2	67 (16.1)
3	152 (36.5)
4	95 (22.8)
5	27 (6.5)
6 and more	19 (4.7)
Missing data	2 (0.5)

^a^Others in ethnicity refers to mixed or other ethnics that not common in Singapore.

^b^Others in employment status refers to individuals who are transiting between jobs.

### 3.2 Main analysis

We first explored the relationship between Hikikomori risk factors (i.e., NHR, *M* = 89.75, *SD* = 19.06) with social withdrawal tendencies (i.e., HQ-25, *M* = 63.20, *SD* = 14.57) and all the related variables (e.g., depression, anxiety, stress, and connection with family and friends). The correlation analysis (Table 2) is presented below.

**TABLE 2:** Bivariate correlations between NHR, HQ-25, connections with family and friends, depression, stress, anxiety, socialization, isolation, and emotional suppression.

	NHR	HQ-25	Family	Friends	Dep	Anx	Str	Soc	Iso	Emo
NHR	–	–	–	–	–	–	–	–	–	–
HQ−25	0.739**	–	–	–	–	–	–	–	–	–
Family^a^	−0.142**	−0.201**	–	–	–	–	–	–	–	–
Friends^b^	−0.412**	−0.538**	0.270**	–	–	–	–	–	–	–
Dep^c^	0.565**	0.560**	-0.128**	−0.353**	–	–	–	–	–	–
Anx^d^	0.407**	0.458**	-0.102*	−0.296**	0.686**	–	–	–	–	–
Str^e^	0.372**	0.394**	-0.099*	−0.271**	0.732**	0.761**	–	–	–	–
Soc^f^	0.681**	0.934**	-0.184**	−0.479**	0.483**	0.392**	0.346**	–	–	–
Iso^g^	0.607**	0.863**	-0.134**	−0.437**	0.514**	0.445**	0.353**	0.715**	–	–
Emo^h^	0.566**	0.687**	-0.196**	−0.459**	0.437**	0.332**	0.306**	0.472**	0.479**	–

*N* = 416. **p < 0.01.

^a^LSNS-family. ^b^LSNS-friends. ^c^Depression levels. ^d^Anxiety levels. ^e^Stress levels. ^f^Socialization (of HQ-25). ^g^Isolation (of HQ-25). ^h^Emotional support (of HQ-25).

With the evidence that Hikikomori risk factors were positively correlated to social withdrawal tendencies and the sub-scales of the HQ-25, we examined the relationship with regression analysis. Results from simple regression analysis showed that high Hikikomori risk factors (IV) predicted high social withdrawal tendencies (DV), *R*^2^ = 0.55, *B* = 0.56, *t* = 22.29, *p* < 0.0011. To have a clearer understanding of how Hikikomori risk factors (i.e., NHR) were connected to each of the HQ-25 sub-scales (i.e., socialization, isolation, and emotional support), we further found that Hikikomori risk factors predicted socialization the best, *R*^2^ = 0.68, *B* = 0.30, *t* = 18.93, *p* < 0.001, and followed by isolation, *R*^2^ = 0.61, *B* = 0.15, *t* = 15.54, *p* < 0.001. NHR predicted less with emotional support, *R*^2^ = 0.57, *B* = 0.11, *t* = 13.98, *p* < 0.001.

Research has suggested that psychosocial and psycho-pathological issues (i.e., primary Hikikomori) and depression, anxiety, and stress (i.e., secondary Hikikomori) could be associated with Hikikomori phenomenon (30). In the correlation analysis, we found that these variables were indeed correlated (either negatively or positively) to Hikikomori risk factors and social withdrawal tendencies, hence, we further examined the relationship in a multiple regression by controlling for primary and secondary Hikikomori. Results found that only depression, *R*^2^ = 0.80, *B* = 0.18, *t* = 2.54, *p* = 0.011, anxiety, *R*^2^ = 0.80, *B* = 0.19, *t* = 2.57, *p* = 0.011, and connection with friends, *R*^2^ = 0.80, B = –1.09, *t* = –7.14, *p* < 0.001 were able to predict social withdrawal tendencies together with Hikikomori risk factors, *R*^2^ = 0.80, B = 0.41, *t* = 14.09, *p* < 0.001.

### 3.3 Additional analysis

**FIGURE 1:**
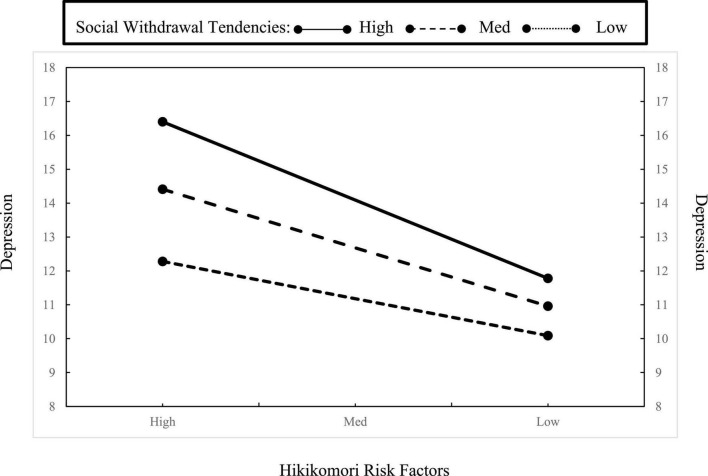
Moderation effect of social withdrawal tendencies on depression within different levels of Hikikomori risk factors.

We further explored the possibility of moderating effects among the issues in the link between Hikikomori risk factors and social withdrawal tendencies. Results suggested that social withdrawal tendencies moderated the relationship between Hikikomori risk factors and depression, *B* = 0.002, *t* = 3.89, *p* < 0.001; specifically, the impact of Hikikomori risk factors on depression was significantly stronger when individuals had higher social withdrawal tendencies (see Figure 1). However, the same moderating effect was not found with other issues (i.e., anxiety, stress, connections from family, and friends), *Bs* < 0.001, *ts* < 2.14, *ps* > 0.05.

We further examined the relationship between emotional regulation strategies and found that individual’s emotional regulation strategies predicted social withdrawal tendencies. Specifically, the more they regulated their emotion *via* suppression (and not for reappraisal) the higher their social withdrawal tendencies, *B* = 1.01, *t* = 7.78, *p* < 0.001.

None of the demographic variables (i.e., employment status, age, gender, number of family members in the household, disability, income, and education levels) showed any significant impact in the relationship between Hikikomori risk factors and social withdrawal tendencies, *Bs* < –0.32, *ts* < 1.67, *ps* > 0.096.

## 4 Discussion

### 4.1 Key findings

In the present research, we explored the relationship between Hikikomori risk factors and social withdrawal tendencies with psychosocial and psychopathological issues that have been associated with Hikikomori. We obtained four findings: One, Hikikomori risk factors were correlated with social withdrawal tendencies, and the primary and secondary Hikikomori issues. Two, as hypothesized, individuals with higher Hikikomori risk factors had higher social withdrawal tendencies. Three, Hikikomori risk factors predicted social withdrawal tendencies together with connection with friends and individual’s depression and anxiety levels, but did not predict connection with family members nor stress levels after controlling the primary and secondary Hikikomori issues. Last, there was an interaction effect between Hikikomori risk factors and social withdrawal tendencies on depression. The higher the withdrawal tendencies, the stronger the relationship between depression and Hikikomori risk factors.

At the first glance, our results seem logical—individuals who have higher Hikikomori risk factors (from cultural marginalization) would engage in more social withdrawal tendencies (e.g., stay home and not to interact with others), suggesting that Hikikomoris in Singapore may follow the Japanese model (5). Interestingly, when we explored further, we found that participants’ depression, anxiety, and lack of connectedness to friends were also possible contributors toward social withdrawal tendencies. Indeed, other studies have found that socially withdrawn Hikikomoris are depressed (6), anxious (27), and have no friends (3). Hence, for individuals who may already be inclined toward Hikikomori risk, these risk factors could further exacerbate their social withdrawal tendencies. However, we found it puzzling that connections to family members did not contribute to the relationship between Hikikomori risk factors and social withdrawal tendencies, which would have been expected from an overview of the literature. We speculated that this could be due to the societal and cultural situation in Singapore. On one hand, Singapore—a traditional Confucian culture, parents feel the need to provide for their children (even if they are emerging adults). Several studies about Singapore parenting styles have found that Singaporean parents adopt the “filial parenting” approach [i.e., parents must take care of their kids to avoid societal judgment; e.g., (39)], mirroring conditions in Japan. However, there is a sharp difference in residence of family units: due to government policy amidst a small land area, individuals in Singapore below age 35 are not allowed to own any public housing. This policy creates a cultural norm where most emerging adults—by default—stay with their family members (parents) at least up to the age of 35. In many situations, they share rooms with their siblings and are unable to avoid family interactions. Therefore, even if they do not feel connected to family, physically distancing themselves would be a comparatively difficult task (i.e., to hide away from family members), hence creating a possible floor effect regarding the connectedness to family members.

In examining psychopathological factors for Hikikomori, we only found that depression was linked to social withdrawal tendencies, and this was more pronounced in individuals with high Hikikomori risk factors. This suggests that the psychopathological risk of Hikikomori (i.e., depression) may be especially pronounced for individuals who are inclined toward withdrawal. It is thus notable that NHR measures one’s inclination toward alternative NEET lifestyle preferences and one’s internalization of being culturally marginalized (e.g., low self-esteem), as these represent areas that may be overlooked during the diagnosis and treatment of Hikikomori individuals. Yet, this may be predictive of potentially stronger risks of depression, suggesting that NHR could represent some antecedent of the maladaptive (depression) aspects of the Hikikomori construct that are not easily picked up by behavioral measures.

Finally, while several past research has found that gender could be a predictor for the Hikikomori phenomenon [e.g., (32, 40)], in our study we did not find any gender differences. However, we noticed that emotion regulation *via* suppression may be problematic for Hikikomori individuals (i.e., high Hikikomori risk factors individuals). Specifically, they might be suppressing their emotional expressions, perhaps, during situations where they feel isolated, depressed, anxious, or disconnected from others, to appear functional in society. For a collectivistic society, this might be a way for them to conform and not “stand out” from the crowd. Liew et al. (5) also identified expression suppression as a strong predictor of NHR in Singapore, but to our knowledge such a relationship has not been observed in Japan or elsewhere. As habitual use of emotion suppression is linked to low trait authenticity (41), which is also linked to reduced wellbeing (42), our concern is that this may be catalyst toward more pronounced social withdrawal tendencies.

### 4.2 Limitations and future directions

Due to the cross-sectional survey design, the analysis (e.g., moderation analyses) in the current study was based on an explorative approach. Hence, our results merely suggests that the relationship between Hikikomori risk factors and social withdrawal tendencies *might be* contributed by depression, anxiety, and the lack of social connections with friends. The moderation between Hikikomori risk factors and depression by social withdrawal tendencies might not be exclusive. An alternative interpretation could be that social withdrawal tendencies predicted depression and is moderated by Hikikomori risk factors. Therefore, we would like to emphasize that the current study is not proposing a causal relationship between these variables. However, we believed that these analyses offered a possible explanation based on existing theory.

In the same vein, due to the lack of theoretical support and existing models to explain the Hikikomori phenomenon in Singapore, we could only explore a few possible (and rational) models in the present study. Therefore, we could have missed the analysis of some important alternative models such as the inclusion of mediational analysis (or other moderators) in our studies (etc., relational mobility, psychological needs, self-construal). Past studies have also shown that risk factors such as low self-esteem, academic difficulties, mental health issues such as avoidant personality disorders and autism spectrum disorders, and gaming and internet addictions would lead to social withdrawal tendencies in other cultures (2, 14, 31). Hence, further studies could assess if these risk factors are mediators or moderators in Singapore through experimental or longitudinal designs.

As mentioned earlier, it was unexpected but interesting that connection with the family did not mediate the relationship between Hikikomori risk factors and social withdrawal tendencies. One limitation could be the use of LSNS-6 as the measurement of connection with family. In some items, the participants might have interpreted the term “relatives” as extended family rather than immediate family. Therefore, the results we obtained regarding social support from family not being a mediator may not be an accurate reflection. For future studies, it might be more reasonable to use the Julkunen Family Support Scale as it measures specifically connection with family members who live with the respondents (43), which may be more in line with actual accessibility of familial interactions for socially withdrawn individuals.

The study was conducted at the initial stage of COVID-19 pandemic and Singapore went into lockdown on Apr 7, 2020. Many of the participants were recruited during the lockdown period in which they were forced to stay home and only allowed to leave their house for essential activities (e.g., getting food). The impact of lockdown due to COVID-19 pandemic was not only physical, but it was also psychological. Studies have suggested that due to social isolation (from the COVID-19 pandemic), comparing to pre-pandemic, around the world (and in Singapore) people scored lower in their subjective and psychological wellbeing in 2020. Further, during the COVID-19 pandemic, individual’s technological and electronical usages increased in frequency (e.g., zoom conference). These resulted in individuals having lesser quality sleep and lack of cognitive resources to stay task-focused and avoid distractions. The overuse of technology during the COVID-19 period had direct and indirect impacts on our health and wellbeings. Hence, some aspects of our results regarding social withdrawal tendencies could potentially be attributed to the COVID-19 pandemic and the lockdown. Therefore, it would be meaningful if future research can replicate the current study to ensure that our results were not solely due to the impact of the COVID-19 pandemic.

Another caveat would be that the demographic of our sample is specific to emerging adults who were between 18 and 35 (34). in the Singaporean context. As mentioned, due to the social and political structure of Singapore, not all emerging adults have the ability (or possibility) to obtain their own place to stay and/or their own room to withdraw to. While our focus was not on actual Hikikomori cases, but on its potential prevalence, we note that it might be difficult to accurately examine actual Hikikomori tendencies in Singapore. Our study used the NHR scale and HQ-25 to measure Hikikomori phenomenon on emerging adults in Singapore to look at associations across scales as constructs. However, we had very few individuals that were above the threshold for high-risk Hikikomori individuals (for NHR, 93 participants out of 416 were above the cut-off at 104; for HQ-25, only 6 participants out of 416 were above the cut-off at 100) (18). As such, our data comprises Hikikomori risk factors and withdrawal tendencies in the general Singaporean population, with only a handful of “actual Hikikomori individuals” in our sample. Nevertheless, we hope that our results serve as foundation for future researchers who want to examine the Hikikomori phenomenon in Singapore.

### 4.3 Interventions

Based on the multidimensional Therapeutic Approaches that consider the physical and social circumstances of Hikikomoris (44), individuals should receive different forms of interventions based on their living status (i.e., living with family members or alone). In Singapore, many individuals (below age 35) live with their family members due to governmental policies. Hence, holistic interventions, such as allowing social workers or clinicians to visit the Hikikomori individual’s home as a form of social support, or educating the family members how to interact with their Hikikomori family member, may be more feasible in the local context. For individuals who might be living alone (i.e., age 35 and above), clinicians and psychologists can use technology (e.g., zoom meeting) to provide web-based therapy or conduct frequent home-visit with the Hikikomori individuals to strengthen their connection to the community.

In Singapore, the Compulsory Education Act and National Service Act require individuals from 6 to 15 years old to attend school and Singaporean males to serve in the armed forces when they reach the age of 18. If individuals (i.e., child or youth) have high Hikikomori risk factors and poor emotional regulation strategies (i.e., high in emotional suppression), these compulsory acts could perpetuate and manifest into other type of psychopathologies, which include non-overt social withdrawal tendencies unique to the Singaporean society. Hence, identifying children or youths who have high Hikikomori risk factors could possibly help to prevent further damage to the individual (psychologically and physically) and the society.

## 5 Conclusion

Social withdrawal not only affects the individual, but it also affects their loved ones and impacts the society (45). Studies have showed that individuals with who socially withdraw, besides having higher risks of health issues [e.g., cardiovascular diseases and stroke, (2); poor quality of sleep, (46)], place a huge burden on caregivers, leading to burnout from, specifically, family members (44). Our results suggested some possible factors behind Hikikomori tendencies, such as connection with friends, depression, and emotion expression suppression, which clinicians and social workers could perhaps tap into in mitigating social withdrawal tendencies as a probable antecedent of actual withdrawal behavior for those at risk of becoming a Hikikomori. As this is a new area of research in Singapore, we invite future studies to use alternative study designs and assessment to measure Hikikomori phenomenon more comprehensively in Singapore. Future studies could also assess other risk factors such as low self-esteem, academic difficulties, mental health issues such as avoidant personality disorders and autism spectrum disorders, and gaming addiction as variables for a holistic view of Hikikomori risk factors and social withdrawal tendencies in Singapore.

## Data availability statement

The original contributions presented in this study are included in the article/supplementary material, further inquiries can be directed to the corresponding author.

## Ethics statement

The studies involving human participants were reviewed and approved by the James Cook University, HREC. The patients/participants provided their written informed consent to participate in this study.

## Author contributions

PL drafted the manuscript and analyzed the data. Andrew applied the ethics application, created Qualtrics, and collected the data under the tutelage of PL. AK initiated the project. AK and KL commented and edited the manuscript. All authors contributed to the article and approved the submitted version.
